# Robocasting of Bioactive SiO_2_-P_2_O_5_-CaO-MgO-Na_2_O-K_2_O Glass Scaffolds

**DOI:** 10.1155/2019/5153136

**Published:** 2019-04-11

**Authors:** Francesco Baino, Jacopo Barberi, Elisa Fiume, Gissur Orlygsson, Jonathan Massera, Enrica Verné

**Affiliations:** ^1^Department of Applied Science and Technology (DISAT), Politecnico di Torino, Turin, Italy; ^2^Department of Mechanical and Aerospace Engineering (DIMEAS), Politecnico di Torino, Turin, Italy; ^3^Department of Materials, Biotechnology and Energy, Innovation Center Iceland (ICI), Reykjavik, Iceland; ^4^Faculty of Medicine and Health Technology, University of Tampere, Tampere, Finland

## Abstract

Bioactive silicate glass scaffolds were fabricated by a robocasting process in which all the movements of the printing head were programmed by compiling a script (text file). A printable ink made of glass powder and Pluronic F-127, acting as a binder, was extruded to obtain macroporous scaffolds with a grid-like three-dimensional structure. The scaffold architecture was investigated by scanning electron microscopy and microtomographic analysis, which allowed quantifying the microstructural parameters (pore size 150–180 *μ*m and strut diameter 300 *μ*m). In vitro tests in simulated body fluid (SBF) confirmed the apatite-forming ability (i.e., bioactivity) of the scaffolds. The compressive strength (around 10 MPa for as-produced scaffolds) progressively decreased during immersion in SBF (3.3 MPa after 4 weeks) but remains acceptable for bone repair applications. Taken together, these results (adequate porosity and mechanical strength as well as bioactivity) support the potential suitability of the prepared scaffolds for bone substitution.

## 1. Introduction

Bone substitution in critical- and medium-sized defects, resulting from injuries, degenerative pathologies, and tumor removal, is still considered one of the major clinical challenges of our time. Hence, there has been a considerable increase in the demand for bone grafts over the last years [1]. Autologous bone is still considered the “gold standard” in realizing bone grafting procedures mainly due to its excellent biocompatibility, but the need to overcome the limits related to tissue availability and donor site morbidity is driving researchers towards other options. Allografts and xenografts are available in a virtually unlimited amount but can carry the risk of disease transmission, are often resorbed more quickly as compared to the host bone healing rate, and may be refused by the patient due to ethical or religious concerns [2]. Hence, many efforts have been carried out in the last two decades for the development of engineered tissues created by bioreactors [3] as well as synthetic bone grafts exhibiting osteoconductive, osteoinductive, and osteogenetic properties [4–6].

Up to now, among all the available synthetic materials used in the production of bone grafts (i.e., polymers, ceramics, and composites [7]), undoubtedly bioactive glasses (BGs) show highly attractive properties as basic materials for the fabrication of three-dimensional (3D) scaffolds based on bone tissue engineering (BTE) strategies [8, 9]. The suitability of BGs relies on their unique capability to both create a stable material-device interface by inducing the formation of a hydroxyapatite (HA) layer on their surface and promote the expression of osteogenetic factors by osteoprogenitor cells [10, 11].

When designing scaffolds for BTE, interconnected pores with mean diameter ≥100 *µ*m and open porosity ≥50 vol.% are considered to be the minimum requirements to have a proper tissue ingrowth and cell migration within the grafting material [12–14]. Moreover, achieving a suitable mechanical response of the device is essential in order to guarantee both structural integrity and adequate support over the whole duration of the healing process [15]. Unfortunately, almost all the traditional manufacturing processes, deeply reviewed elsewhere [16–18], do not allow achieving an accurate control on such parameters.

As safety and reliability of the implant-manufacturing process represent moral imperatives in health sciences, methods for the production of complex geometries and patient-specific devices are strongly required. As a result, starting from the early '80s, solid freeform fabrication (SFF) techniques, also known as additive manufacturing (AM) technologies, gained increasing scientific interest because of the possibility to easily tailor the device properties by simply acting on process parameters [19, 20]. Such technologies, in fact, are based on bottom-up approaches, where the object is produced layer by layer starting from a computer-aided design (CAD) file (.stl file) or text scripts [15]. All these aspects are of particular interest in BTE where obtaining customized devices is a primary goal [21]. A high control on porosity, pore size, and interpore interconnectivity can actually be achieved by relatively simple SFF processes. These techniques show also promise for large-scale manufacturing, where high reproducibility of the devices is required.

Moreover, having a high control on the scaffold architecture makes it possible to tailor the mechanical response of the device during the design phase [22]. In this regard, it should be pointed out that both compressive strength and elastic modulus of the bone should ideally be matched by the scaffold. These parameters depend on both the extruded material properties and the scaffold pore/strut structure.

Several SFF techniques have already been used for BTE scaffold fabrication, including 3D printing (3DP), fused deposition modeling (FDM), ink-jet printing, stereolithography (SL), and selective laser sintering (SLS) [20]. However, very little information is available in the literature regarding the processing of bioactive glasses by SFF techniques. Probably, robocasting is the most common and powerful direct ink-writing technique for the processing of glass and glass-ceramic scaffolds. This technique is based on the continuous extrusion of a filament (ink) from a robot-controlled nozzle onto a building platform [22]. The ink is a slurry, composed of glass or ceramic particles and a polymeric binder to form a colloidal suspension, characterized by well-defined rheological properties [23]. Usually, the process does not require the use of a high concentration of binder, allowing sintered parts to be obtained in a short time [22]. Pluronic F-127 is one of the three most commonly used binders for robocasting in bone applications [24–26], together with ethyl cellulose/polyethylene glycol and carboxymethyl cellulose [27–30].

Robocast bioceramic scaffolds were produced for the first time in 2010 by Franco et al., who developed a hydrogel-based ink containing calcium phosphates (HA and *β*-TCP) [24]. Since then, both commercial 45S5 Bioglass® and 13-93 glass were processed by robocasting by several research groups. In 2013, Liu et al. used robotic deposition to produce 13-93 glass-based grid-like microstructured scaffolds with 47 vol.% porosity and 300 *µ*m pore width. Flexural and compressive mechanical tests were performed before and after both bioactivity tests in simulated body fluid (SBF) and in vivo tests in a rat subcutaneous model. It was found that compressive strength decreased both after 2-week immersion in SBF and in vivo implantation. Moreover, a shift from brittle to elastoplastic response was observed after 2- and 4-week implantation in vivo, thus demonstrating the bone-like behaviour of such devices and their suitability in load-bearing applications [26].

In 2014, fully vitreous 45S5 Bioglass®-based scaffolds with interconnected porosity ranging from 60 to 80 vol.% were successfully produced for the first time. All the scaffolds showed compressive strength comparable to that of the trabecular bone (2–13 MPa), even when sintered below the crystallization temperature [27].

More recently, 45S5 Bioglass®-based scaffolds reinforced by HA/PCL nanocomposite coatings were obtained by Motealleh et al. [30], who interestingly investigated the effects of different postprocessing thermal treatments on the scaffold mechanical response. The CAD-derived original architecture was successfully retained upon sintering both in amorphous and highly crystallized scaffolds, with compressive strengths of 2 MPa and 11 MPa, respectively, which are definitely in the range of the trabecular bone.

In a recent study, functionally graded porous devices were successfully obtained by Mattioli-Belmonte et al. [31] who used a robocasting system, called the pressure-assisted microsyringe (PAM), to produce bioactive glass/poly(lactic-co-glycolic acid) (PLGA) 2D porous structures characterized by a well-defined topology. The layers were then assembled in order to obtain a 3D bone-like scaffold [31]. It was demonstrated that the elastic modulus was comparable to that of the cancellous bone, and osteoblastic differentiation of human periosteal precursor cells was observed, showing great promise for bone tissue engineering applications.

In the present work, highly bioactive and fully amorphous grid-like scaffolds for bone regeneration were produced by robocasting using a six-oxide silicate glass as the basic material for the ink formulation. The aim was to demonstrate that it is possible to obtain 3D structures with suitable porosity with a rather simple method, avoiding the use of ultrafine powders, thin nozzles, and complex ink preparation.

## 2. Materials and Methods

### 2.1. Glass Preparation

The basic material used to manufacture the scaffolds was a silicate glass (composition 47.5SiO_2_-10Na_2_O-10K_2_O-10MgO-20CaO-2.5P_2_O_5_ mol.%) originally developed by Verné et al. [32] at Politecnico di Torino. This glass, referred to as 47.5B, was produced by a standard melting method in a platinum crucible. The raw precursors (SiO_2_, Na_2_CO_3_, K_2_CO_3_, (MgCO_3_)_4_·Mg(OH)_2_·5H_2_O, CaCO_3_, and Ca_3_(PO_4_)_2_ high-purity powders purchased from Sigma-Aldrich) were homogeneously mixed in the crucible and melted in air at 1500°C for 30 min. The melt was then quenched in deionized water to produce a frit that was ball-milled (Pulverisette 0, Fritsch, Germany) and sieved to obtain a final particle below 32 *μ*m by using a stainless steel sieve (Giuliani Technologie Srl; mesh 32 *μ*m).

### 2.2. Scaffold Fabrication by Robocasting

The 47.5B glass was thought to be very suitable to produce completely amorphous—and hence highly bioactive—porous scaffolds due to its large hot-working range (difference between onset of crystallization (*T*_x_) and glass transition temperature (*T*_g_), *T*_x_ − *T*_g_ = 260°C), as determined in a previous study [32].

Pluronic F-127 (Sigma-Aldrich) was used as a binder for the preparation of the glass-based ink. The ink formulation was optimized through some preliminary trials and involved the addition of 35 vol.% of glass to an optically clear water-based solution containing 27.5 wt.% of Pluronic F-127. Prior to adding the glass, the Pluronic F-127 solution was stirred overnight while being maintained at low temperature in an ice bath due to the thermosensitive behaviour of the binder. The glass-containing ink was then mixed for 1 min by using a vortex mixer (Ika-Werk shaker, type Vibrofix VF1 electronic) at 2500 rpm and cooled for 1 min in the ice bath. Five mixing-cooling cycles were performed to allow achieving good dispersion of the glass particles.

The plastic cartridge connected to the robocasting machine (3Dn-Tabletop, nScrypt Inc., Orlando, FL, USA) was filled with the ink, which was left to stabilize for 1 h before printing. The only movement that was allowed to the printing tower was along the (vertical) *z*-axis, and its position determined the printing height. Plastic tips with an inner diameter of 410 *μ*m (Nordson EFD Optimum® SmoothFlow™) were used to extrude the ink. The plate under the nozzle was moved on the *x-y* plane with respect to the printing tower so that the ink was extruded according to the correct pattern. The printing accuracy was 10 *μ*m along the *x*- and *y-*axes and about 5 *μ*m along the *z*-axis [33].

Acetate sheets (Colour Copier and Laser Transparency OHP Film, Folex AG, Seewen, Switzerland) were used as the printing substrate due to their flatness, good adhesions they have with the ink, and easiness to detach the scaffolds from them once they are dry [34].

**Figure 1:**
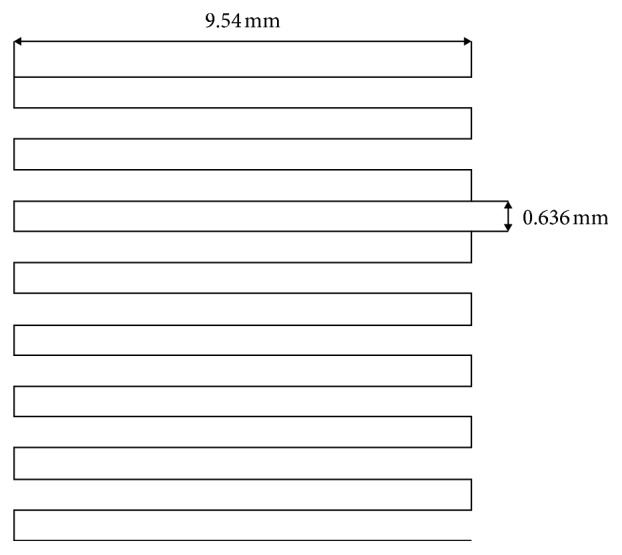
Raster pattern used to print the scaffolds.

Once the cartridge was loaded and fitted in position and the acetate sheet was placed on the platform, the processing parameters were adjusted using the software (MachineTools 3.0) provided by nScrypt. The desired structure was obtained by programming every single movement that the print head must do along the *x*-, *y*-, and *z*-axes through compiling a design script, written as a text file. The printing speed was 2 mm/s, and the pressure used to extrude the ink was in the range of 1.24–1.51 bar; the raster pattern is shown in Figure 1.

Robocast 47.5B scaffolds were porous cuboids (length = width = 7.5 mm) with a grid-like structure and were made of 20 glass layers (height about 4.5 mm). Once the printing was completed, the scaffolds were left to dry for 48 h in air and finally detached from the acetate sheet. A multistep thermal treatment (three stages at 200, 400, and 500°C for 30 min each followed by a final stage at 600°C for 1 h; heating rate 1°C/min) was eventually performed to allow the removal of the organic binder and the sintering of glass particles. Only the last heat treatment, being performed at a temperature higher than *T*_g_, led to glass sintering, while the others were part of the burning-out process of the binder, which was carried out slowly to ensure the complete removal of any organic residue and avoid cracking phenomena due to sudden shrinkage.

### 2.3. Characterizations

#### 2.3.1. X-Ray Diffraction

Both as-quenched glass and sintered scaffolds (after being crushed into powder) underwent wide-angle X-ray diffraction (XRD; 2*θ* within 20–70°) to detect the presence of crystalline phases. A X'Pert Pro PW3040/60 diffractometer (PANalytical, Eindhoven, Netherlands) was used; the experimental setup included operating voltage 40 kV, filament current 30 mA, Bragg-Brentano camera geometry with Cu K*α* incident radiation (wavelength *λ* = 0.15405 nm), step size 0.02°, and fixed counting time per step 1 s.

#### 2.3.2. In Vitro Bioactivity

The bioactivity of the robocast scaffolds, in terms of HA formation and ionic release in vitro, was carried out by properly adapting the testing procedure proposed by Macon et al. [35]. The experiments involved the immersion of triplicate samples in Kokubo's SBF [36] for 6, 24, 48, 72, 168, and 336 h. The ratio between sample mass and SBF volume at the beginning of the experiments was fixed at 1.5 mg/ml. The specimens were placed into an orbital shaker incubator (Multitron AJ 118 g, Infors, Bottmingen, Switzerland) at 37°C under a constant speed of 100 rpm. The solution was analyzed at each time point by means of inductively coupled plasma optical emission spectroscopy (ICP-OES) (5110 ICP-OES, Agilent Technologies) in order to evaluate the concentration of ions in the solution. After being extracted from the solution, the samples were gently rinsed with distilled water and left to dry overnight at room temperature. The results were expressed as mean ± standard deviation.

#### 2.3.3. Morphology and Porosity

The scaffolds were investigated before and after in vitro tests by a field-emission scanning electron microscope (FE-SEM; Supra™ 40, Zeiss, Oberkochen, Germany) equipped with an energy dispersive spectroscopy (EDS) detector in order to evaluate the pore-strut morphology and the formation of new phases on the surface of SBF-treated samples. The specimens were sputter-coated with chromium prior to the analysis and inspected at an accelerating voltage of 15 kV.

The total porosity of the scaffolds was assessed by mass-volume measurements as (1 − *ρ*/*ρ*_0_) × 100, where *ρ* is the apparent density of the scaffold and *ρ*_0_ is the bulk density. The porosity was expressed as mean ± standard deviation calculated on five specimens.

For micro-CT analysis, the samples were X-ray scanned in the dry state in a Phoenix Nanotom S machine (General Electric Measurement and Control), at a source voltage of 110 kV and a source current of 110 *µ*A. No X-ray filters were used. The scanning modalities for the robocast 47.5B scaffolds before and after immersion in SBF are reported in Table 1. A translational motion compensation was performed in order to guarantee a perfect matching of the 0*°* and 360° shadow images. Thereafter, the projection images were used to reconstruct the investigated scaffolds by means of the Radon transform [37, 38] as algorithmized in the software datos|x reconstruction provided by the manufacturer. VGStudio Max 2.0 from Volume Graphics was employed for visual evaluation and detail measurements on strut and void sizes as well as for calculations of porosity. An automated calibration routine integrated into VGStudio Max 2.0 was used to define material boundaries. The software determines the background peak and the material peak in the grey value histogram and calculates the grey value of the material boundary. VGStudio Max 2.0 was also used to export image stacks in the DICOM format. This format can be read into the BoneJ plugin [39] running under the ImageJ software package (version 1.51t) [40]. BoneJ (version 1.4.2) was used to extract information following an approach used for the trabecular bone where bone volume, total volume, trabecular thickness, and trabecular spacing (i.e., pore size) are determined [41].

#### 2.3.4. Mechanical Characterization

The compressive strength of the scaffolds before and after in vitro tests (2 and 4 weeks in SBF) was evaluated through crushing tests by using an MTS machine (Model 43, MTS, Minnesota, USA; cell load 5 kN and cross-head speed 1 mm·min^−1^). The failure stress was calculated as the ratio between the maximum load registered during the test and the resistant cross-sectional area measured by callipers. The compressive strength was expressed as mean ± standard deviation calculated on five specimens for each type.

## 3. Results and Discussion

### 3.1. Microstructure and Morphology

**Figure 2:**
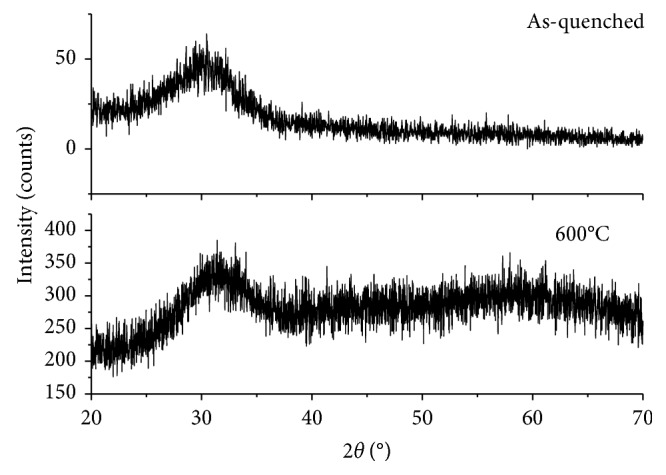
XRD patterns. Both the as-quenched 47.5B glass powders (red) and the powdered scaffolds sintered at 600°C for 1 h (black) are characterized by the amorphous structure since no crystallization peaks were observed.

Figure 2 shows the XRD spectra of both the as-quenched material and the sintered scaffolds. As expected, no diffraction peaks can be detected, but only a broad amorphous halo in the range of 20 to 35° is visible, which is typical of silicate glasses (red pattern). No microstructural changes in the material were revealed after the thermal treatment at 600°C, which demonstrates that 47.5B scaffolds remain in an amorphous state (black pattern). This is beneficial for the bioactivity, as suggested by some studies reporting that devitrification can reduce the apatite-forming ability of bioactive glass-derived materials [42].

**Figure 3:**
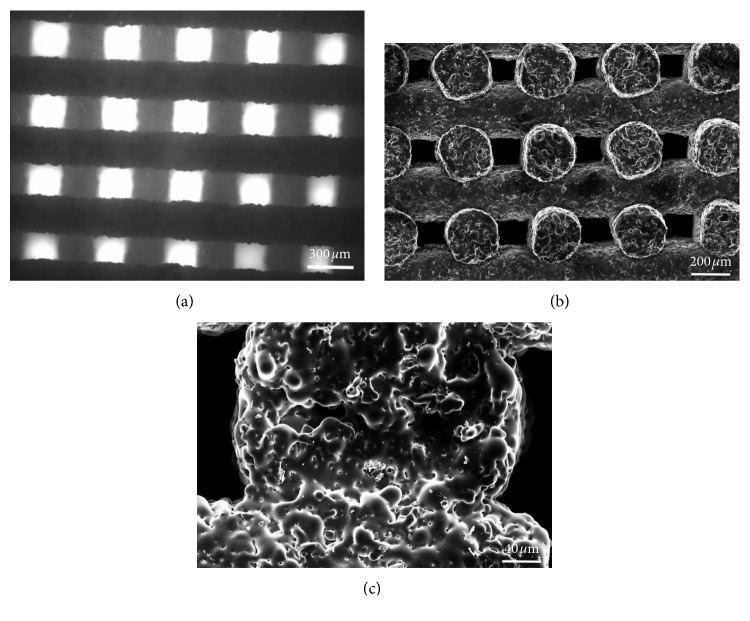
Morphological evaluation of the scaffold. Top view of the grid-like structure obtained by optical microscopy (a); SEM micrograph of the channel porous structure (b) and of the trabecular section (c).

The scaffold structure obtained by robocasting is highly regular with a grid-like arrangement of the pores (Figure 3(a)). The struts (rods) remained straight during the sintering process and exhibited a quite regular circular section. The porosity is made of regular macrochannels, both vertical and horizontal, generated by the separation between glass lines and the tilting of each newly juxtaposed layer with respect to the underlying one.

More accurate morphological investigations were carried out by SEM, especially in order to understand the level of sintering reached during the thermal treatment. The scaffolds exhibit well-densified struts (rods) in which the glass particles are no more distinguishable (Figure 3(b)). High-magnification analysis reveals that the interparticle porosity almost completely disappeared as a result of sintering and the glass particles are fused together, also in the areas of contact between adjacent rods (Figure 3(c)). Few small spherical pores derived from air bubbles entrapped in the ink can also be observed. The cross-sectional size of the channels (100–200 *μ*m) is potentially suitable to support new bone formation. In fact, pores above 100 *μ*m allow osteoblastic cell colonization and proper vascularization, thus avoiding hypoxic growth conditions of the bone [9].

The total porosity of the scaffolds was 42.5 ± 4.5 vol.%, which is close to the minimum threshold of acceptability recommended for bone tissue engineering applications (about 50 vol% [43]). Further optimizations of the robocasting process could allow increasing the porosity without negatively affecting the structural integrity. The low value of standard deviation demonstrates the good reproducibility of the fabrication process.

**Figure 4:**
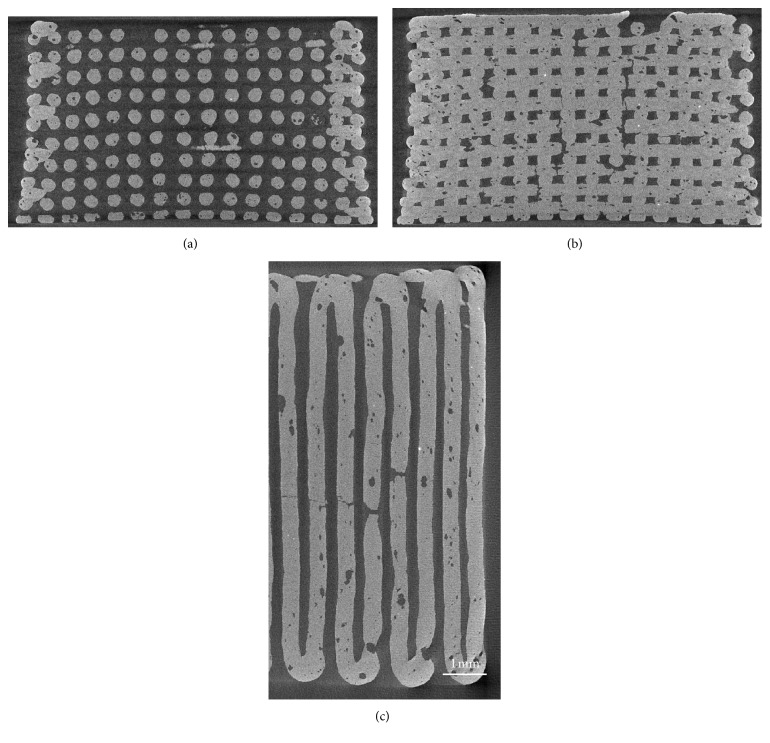
Micro-CT images of 47.5B scaffolds. Vertical section on a plane that passes through the gap between 2 central rods (a); vertical section on a plane that cuts through the rods parallel to the image plane (b); horizontal section on the midheight of the scaffold, showing also the border of the scaffold (c) (nominal strut diameter: 300 *μ*m).

Micro-CT reconstructions of two-dimensional sections of the scaffolds are reported in Figure 4.

The tomographic images showed good regularity of the pore-strut structure and rod diameter: the voids that are visible in the filaments are due to air bubbles that remained entrapped inside the ink. In some cases, these voids originated from full-thickness cracks in the filaments as a consequence of the scaffold volumetric shrinkage upon sintering. Quantification of microstructural parameters yielded the following results: pore width 180 ± 25 *μ*m, pore height 147 ± 19 *μ*m, and strut diameter 300 ± 10 *μ*m. These findings are consistent with those from SEM observations; the low values of standard deviation confirmed the good reproducibility of the robocasting process.

A slight curvature in the rods of the scaffold structure is visible in Figure 4(a). This could be due to (i) the slight bending of the ink filaments under their own weight prior to sintering and (ii) shrinkage phenomena that occur during sintering.

**Figure 5:**
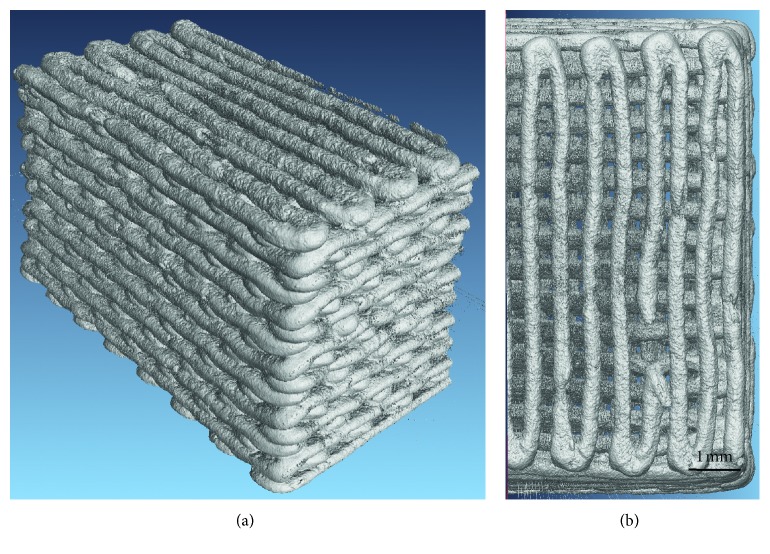
3D reconstruction of scaffold volume obtained by micro-CT. Lateral view (a); top view (b) (nominal strut diameter: 300 *μ*m).

Three-dimensional reconstructions of the whole scaffold volume are also displayed in Figure 5.

### 3.2. In Vitro Bioactivity

**Figure 6:**
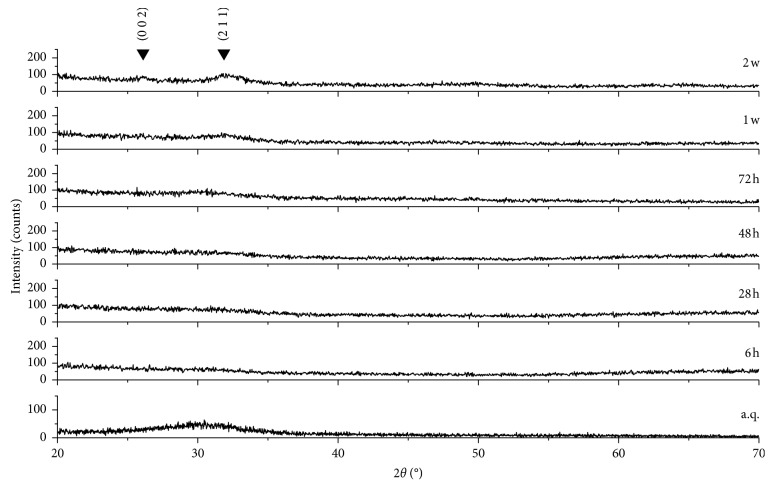
XRD spectra of 47.5B scaffolds after different immersion times in SBF. The characteristic peaks of HA are indicated by Miller indices (*h k l*).

A biomaterial for bone repair is defined “bioactive” if HA precipitates onto its surface once it is implanted inside a living body or during immersion in solutions that simulate the body environment (in vitro) [44]. XRD analyses on the samples soaked in SBF for different time frames actually revealed the formation of HA on the scaffold surface (Figure 6).

Specifically, there was a progressive disappearance of the amorphous halo typical of the glass and the appearance of the HA characteristic diffraction peaks. The two main peaks of HA (PDF code no. 01-073-1731) were detected, the highest at 31.79° (corresponding to the (2 1 1) reflection) and the other one at 25.68° ((0 0 2) reflection). These peaks are not sharp but have a quite broad appearance, suggesting that the HA formed on the scaffold struts has a nanocrystalline nature.

**Figure 7:**
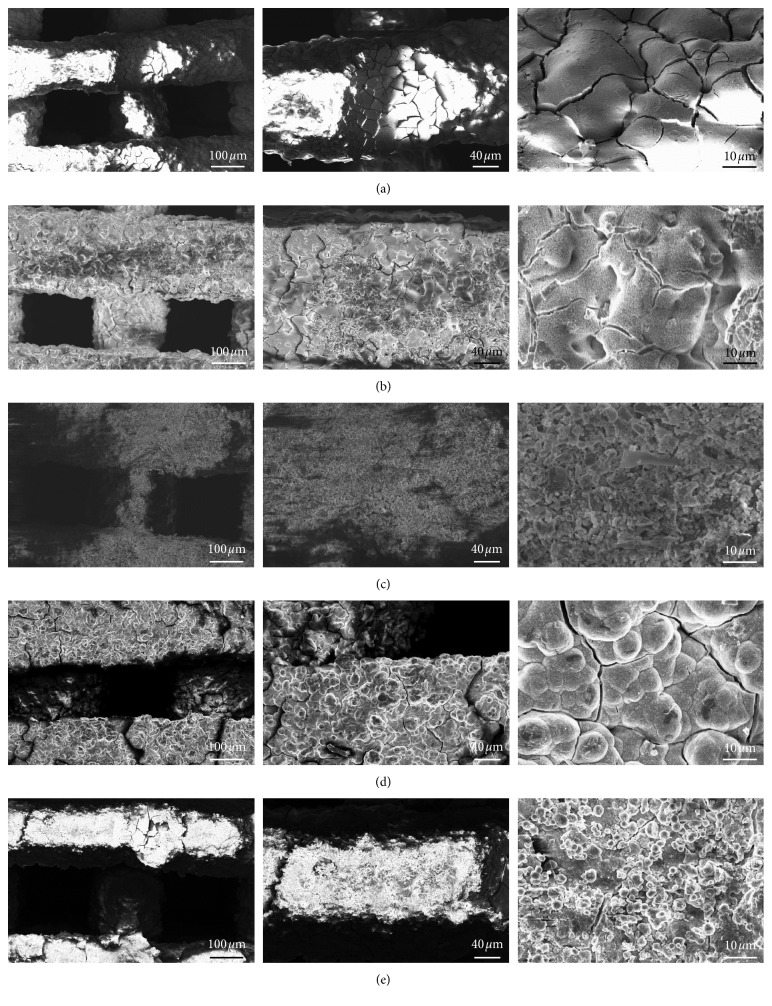
SEM morphological analysis performed after immersion in SBF at different time points. Typical silica gel layer observed on the surface of the material after soaking for 6 h (a); small calcium-phosphate nuclei formed after 24 h (b); gel layer evolution upon soaking observed after 72 h (c); formation of a thick layer of globular HA characterized by the typical cauliflower morphology after 1-week immersion (d); morphology evaluation of the surface after 2 weeks revealed a partial detachment of the HA with no significant changes in the rod diameter (e).

A better understanding of the in vitro bioactivity mechanism of 47.5B scaffolds was obtained performing morphological and compositional analyses by SEM and EDS assisted by micro-CT imaging. Thus, it was possible to follow the stepwise evolution of the scaffold surface, including the formation of the silica gel, the precipitation of the amorphous calcium-phosphate layer, and the nucleation and growth of HA crystals, according to the bioactivity mechanism proposed by Hench [45]. After 6 h, it is possible to see the formation of the silica gel (the typical surface-cracked layer in Figure 7(a)), and at 24 h, small calcium-phosphate nuclei are visible as brighter globules on the surface of the underlying gel (Figure 7(b)). This layer evolved leading to the formation of needle-like HA crystals that grew and joined together to form large globular crystals with “cauliflower morphology,” which is typical of bone-like HA (Figures 7(c)–7(e)). After 1 week, the scaffold struts are thicker as compared to those of untreated scaffolds and are covered by HA globular aggregates. After 2 weeks, no significant thickening of the scaffold rods is observed, suggesting that a process of HA detachment from the scaffold might have taken place. This is evidenced by the fact that the HA crystals detected on the scaffold walls are smaller compared to the ones that covered the 1-week specimen, as it is possible to see in Figure 7(d). The Ca-to-P atomic ratio increases with soaking time from 0.46 at 24 h to 1.47 at 2 weeks. The Ca-to-P ratio at the end of the experiments is still lower that the value of stoichiometric HA (Ca/P = 1.67), revealing the presence of Ca-deficient HA as already observed previously for other bioactive glass compositions [46–48]. However, as Mg^2+^ ions are released from the glass surface during immersion in SBF, a small amount of Ca in HA has been substituted by Mg, as confirmed by compositional analysis. Therefore, the atomic ratio between the amounts of bivalent cations and phosphorus (Ca + Mg)/P yields 1.64, which is closer to the Ca/P value of stoichiometric HA.

**Figure 8:**
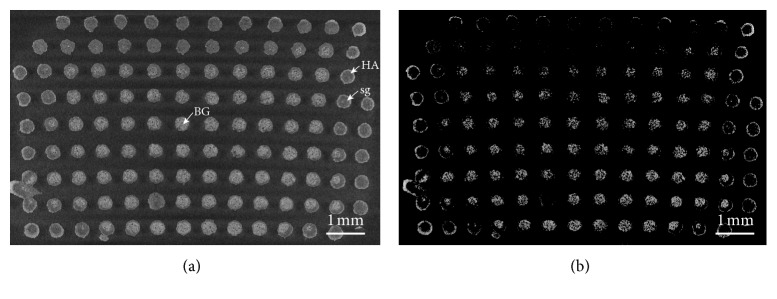
Cross section of a 47.5B scaffold soaked in SBF for two weeks obtained by micro-CT. (a) Standard condition and (b) augmented contrast. HA, hydroxyapatite layer; sg, silica gel; BG, unmodified bioactive glass.

The micro-CT analysis of the scaffold structure after 2 weeks in SBF highlighted the presence of different reaction layers in the rods resulting from the bioactivity mechanism (Figure 8).

Some rods clearly exhibit a thick silica gel layer (grey tones) with a thin converted outer layer of HA (white tones). Other struts, mainly at the scaffold periphery, present a quite thick HA coating and a core converted to silica gel. A third type of rods is represented by the trabeculae in the center of the scaffold which are almost unmodified. HA and bioactive glass, which have higher densities than silica gel, are highlighted in Figure 8(b).

Ions are released from the glass surface during the reaction stages of the bioactivity mechanism; therefore, it is possible to gain some additional information about this process by monitoring the evolution of the ionic concentration in SBF during the dissolution test. In fact, it was previously shown that greater changes in ionic concentrations can be related to higher bioactivity [49].

**Figure 9:**
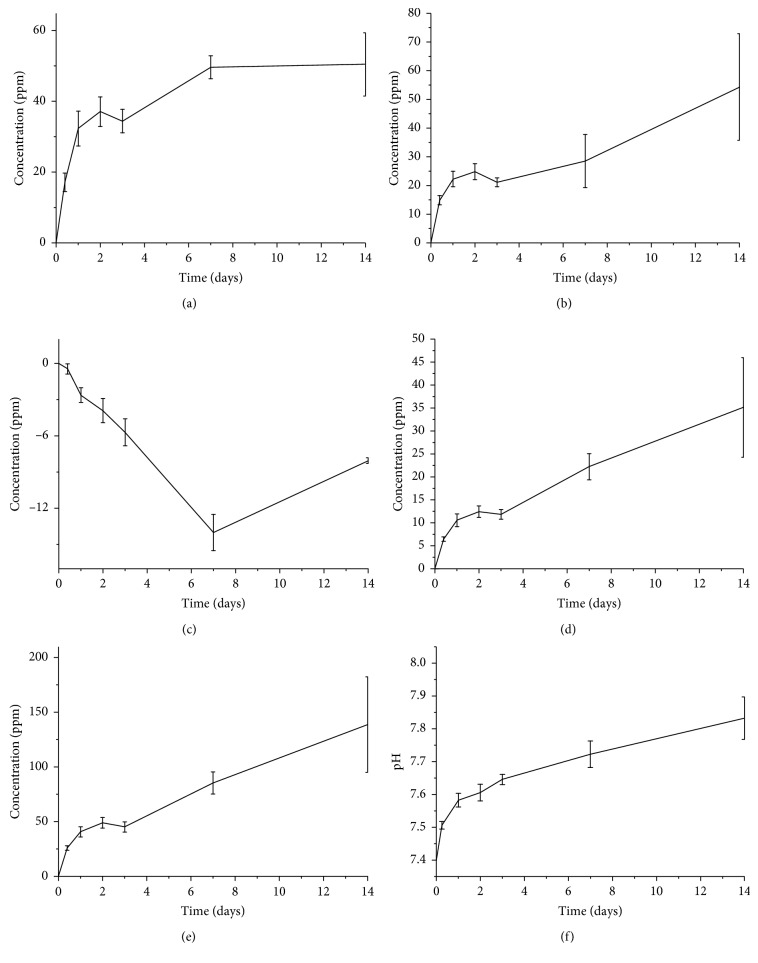
Ion-release profiles related to Si (a), Ca (b), P (c), Mg (d), and K (e) and pH (f) trend upon soaking in SBF. The variation of ionic concentration in the solution is reported after the subtraction of the ion concentration of blank SBF.

Silicon concentration increases steadily over the first week (Figure 9), when the glass converts into silica gel releasing soluble Si(OH)_4_, and reaches the equilibrium at 1 week after the formation of an HA layer. Also, a similar trend was observed for calcium ions within 1 week, but a plateau was not reached.

The trend of phosphorus is indicative of the sequestration of phosphate ions from the solution, which means that calcium phosphate—and then HA—precipitates for the whole soaking period. This is in good agreement with the SEM and micro-CT observations (Figures 6 and 8). The trends of magnesium and potassium ions are in accordance with the evolution of the silicon concentration in the first week, confirming a progressive dissolution of the glass. However, while silicon concentration reaches a plateau after 1 week, the concentrations of magnesium and potassium in SBF still tend to increase over the whole testing period. The evolution of the ionic concentrations of Si, Ca, and P is in agreement with previous studies on the dissolution of bioactive silicate glasses [50]. The release of sodium is not reported because its concentration, extremely high within the SBF solution, oversaturated the detector.

The pH of the solution was also monitored during the test as its variations are strongly related to the ionic exchanges between glass and SBF. The pH values, shown in Figure 9, exhibit a rapid increase in the first 24 h, and then the increase in pH slowed down. This is consistent with the morphological observations of the sample: since the pH is modified by the ionic exchanges between the glass and the solution, once the HA layer starts to form and limit the glass-SBF interactions, the ion exchange rate decreases too.

### 3.3. Mechanical Strength

The compressive strength of as-printed scaffolds was in the range of 5.6–16.5 MPa (9.9 ± 4.6 MPa), which is comparable to the typical range assessed for the human cancellous bone (2–12 MPa [51]). Future optimizations in the process of scaffold fabrication could allow reducing the high variability of the mechanical strength.

The mechanical properties of scaffolds should ideally match those of the host bone, and this is challenging especially in load-bearing applications. The advent of additive manufacturing technologies disclosed fascinating scenarios in this regard. An interesting study carried out by Rainer et al. [52] established the basis for successfully achieving the design of scaffolds with the microarchitecture predicted through a priori finite-element analysis (FEA) of the implant site geometry under physiological loads. This approach, called the load-adaptive scaffold architecturing (LASA), uses FEA for obtaining the principal stress directions under a physiologically derived load system and can easily be coupled to CAD-based manufacturing technologies. Although the applications of LASA are currently limited to polymeric materials, extension to glasses and ceramics would be highly beneficial for the field of BTE scaffolds.

When a scaffold is implanted in a load-bearing site, its major function is to act as a template for the new bone growth while supporting the surrounding tissues, exactly as a healthy bone does. Thus, the mechanical properties of the scaffold need to be suitable at the moment of the implantation and, furthermore, should not decrease too fast during the healing time. The compressive strength of the scaffolds decreases as the immersion time in SBF increases (5.6 ± 2.2 MPa after 2 weeks and 3.3 ± 0.7 MPa after 4 weeks) but still remains within the typical range of the trabecular bone (2–12 MPa [51]). This trend is in agreement with the results reported by Motealleh et al. [30] who used robocasting to fabricate 45S5 Bioglass® scaffolds with a grid-like structure.

## 4. Conclusions

Bioactive glass scaffolds were obtained by a relatively simple robocasting process that does not require the use of ultrafine glass powder, very thin nozzles, and a complex experimental setup, which are usually needed in the processes reported in the literature. The process allowed fabricating macroporous scaffolds with well-reproducible microstructural features, such as pore size and rod diameter. The compressive strength of the scaffolds, which remains comparable to that of the cancellous bone even after prolonged immersion in SBF with ionic dissolution phenomena, combined with a clear apatite-forming capability supports the potential suitability of the material for bone repair applications.

## Figures and Tables

**Table 1:** Micro-CT scanning parameters.

Scaffold	Magnification	Voxel size (*µ*m)	Rotation step (°)	Exposure time (s)	Tube mode	Frame averaging number	Frame skipped
Scaffold as-such	10.00x	5.00	0.50	1.5	0	3	1
Scaffold soaked for 2 weeks in SBF	11.11x	4.50	0.50	1.5	0	3	1

## Data Availability

The data used to support the findings of this study are included within the article.
